# Heavy Rainfall and Flooding Effects on Maternal Health Service Delivery During Pregnancy and Childbirth in LMICs: A scoping review protocol.

**DOI:** 10.3310/nihropenres.14311.1

**Published:** 2026-08-18

**Authors:** Valentine Bahati, Moriasi Nyanchoka, Pauline Karing’u, Conrad Wanyama, Jacob McKnight, Michuki Maina

**Affiliations:** 1 Health Services Implementation Research, KEMRI-Wellcome Trust Research Programme Nairobi, Nairobi, Nairobi County, Kenya; 2 Heath Systems Collaborative, Centre for Tropical Medicine & Global Health, Nuffield Department of Medicine, University of Oxford, Oxford, England, UK

**Keywords:** Climate change, Extreme weather events, Flooding, Heavy rainfall, LMICs, Maternal Health Services, Pregnancy

## Abstract

**Introduction:**

Climate change is a major global health challenge of the 21 ^st^ century associated with the increasing frequency and severity of extreme weather events, including heavy rainfall and flooding. These extreme weather events are particularly disruptive in low- and middle-income countries (LMICs), as they damage infrastructure, impede access to healthcare, and destabilize service delivery. Such disruptions heighten risks of missed or delayed antenatal care, reliance on unskilled obstetric care, and interruptions in postpartum care, contributing to adverse maternal and newborn outcomes. Despite growing evidence linking climatic hazards to poor maternal health, the pathways through which extreme weather events, specifically heavy rainfall and flooding affect the delivery of maternal health services remain insufficiently mapped.

**Objective:**

To map and synthesize existing evidence on how heavy rainfall and flooding affect accessibility and quality of maternal health service delivery during pregnancy and childbirth in LMICs.

**Methods:**

This scoping review will follow the Joanna Briggs Institute (JBI) methodology for scoping reviews. We will include quantitative, qualitative, and mixed-methods studies, as well as relevant reports and evaluations. A comprehensive search strategy will be developed in consultation with a research librarian, and implemented in MEDLINE, Global Health, and Web of Science, complemented by relevant grey literature sources, to identify articles published between 1990 and 2025. Screening will be conducted independently by two reviewers using Covidence, with discrepancies resolved through discussion. Data will be extracted using a standardized charting form and synthesized narratively to capture patterns and mechanisms of service disruption.

**Conclusion:**

This scoping review aims to map and synthesize existing evidence on the effects of heavy rainfall and flooding on maternal health service delivery in LMICs. By identifying key patterns, challenges, and gaps in the literature, the review will inform future research and support policy and planning efforts to strengthen climate-resilient maternal health services.

## Plain Language summary

This study explores how heavy rainfall and flooding affect access to and quality of maternal healthcare services in low- and middle-income countries (LMICs). Maternal healthcare includes services such as antenatal care during pregnancy, skilled birth attendance, emergency obstetric care and postnatal care.

Climate change is associated with an increase in the frequency and severity of rainfall and flooding. Particularly in regions with already limited healthcare infrastructure, increased frequency and severity of rainfall and flooding can damage roads, disrupt transport systems, and disrupt the functioning of health facilities, making it more difficult for pregnant women to access timely and quality care.

This research will review already published studies to understand how heavy rainfall and flooding affect maternal healthcare services. It will also explore how health systems are disrupted, including challenges such as damaged infrastructure, shortages of healthcare workers and supplies, and delays in reaching care.

The findings will help identify gaps in current knowledge and support efforts to improve healthcare systems so that they can respond better to climate-related challenges and ensure continuity of maternal care.

## Introduction

### Background and Rationale

Climate change, attributed to the rising global temperatures, is associated with the increasing occurrence of extreme weather events, including heavy rainfall and flooding. ^1^ Heavy rainfall is defined as precipitation exceeding a high local percentile threshold (typically ≥95th percentile of daily totals; very heavy ≥99th percentile) over a given period and geographic area, often overwhelming natural and built drainage systems and increasing the risk of flooding. ^2^ Flooding is an accumulation of drainage water over areas which are not normally submerged, commonly due to prolonged or intense rainfall, river overflow, or inadequate drainage infrastructure. ^3^

Climate-related hazards are seen to greatly affect health and the healthcare systems globally, causing an increase in injuries, displacement, morbidity, and mortality. ^4^ Climate change also impacts the health workforce and health infrastructure, reducing system capacity to deliver universal health coverage (UHC). ^5^ Floods in particular are associated with increased risks of morbidity and mortality globally. Between 1998–2017, floods affected more than 2 billion people worldwide ^6^ and over 200,000 deaths in the past three decades. Although drowning is the leading cause of flood-related deaths, other immediate causes include adverse health outcomes, damage to health facilities, and disrupted access to essential health services. ^7^

Low- and middle-income countries (LMICs) are at a higher risk of disruption of health and health services from heavy rainfall and flooding due to limited infrastructure, poor governance, and inadequate resources to adapt to the effects of these climatic hazards. ^8^ The Intergovernmental Panel on Climate Change (IPCC) estimates that the effect of climatic hazards was 15 times more intense in vulnerable regions (LMICs) than in less vulnerable regions. ^1^ In these settings, heavy rainfall and flooding frequently damage roads and bridges, geographically isolates communities, and restricts movement, making it difficult for both patients and health workers to reach health facilities. ^1^ Health facilities may be damaged or declared non-functional, and there could be a shortage of resources (material and human). These disruptions affect the delivery of maternal health service, a critical time-sensitive health care service. ^9^

Maternal health services involve a range of health services provided to women during pregnancy, childbirth, and the postpartum period, with the goal of ensuring safe outcomes for mothers and newborns. ^10^ These services include antenatal care, skilled attendance at birth, emergency obstetric and newborn care, and postnatal follow-up. Effective delivery of maternal health services depends on several interrelated health system components, including physical access to facilities, availability of skilled health workers, functional referral and transport systems, facility readiness, availability of required resources, and continuity of care across the antenatal, intrapartum, and postpartum periods. ^11^ Disruptions to any of these components can result in delays in care, compromised safety, and increased risk of adverse maternal and perinatal outcomes.

For the purposes of this scoping review, maternal health service delivery is operationally defined as the availability, accessibility, and quality of formal health services provided to women during pregnancy and childbirth. In line with internationally accepted health systems and quality-of-care frameworks, this review focuses on two key dimensions of service delivery that are particularly vulnerable to heavy rainfall and flooding disruptions: access to maternal health services and quality of maternal health services. Access involves the ability of pregnant women and healthcare workforce to physically reach health facilities. The quality of maternal health services will be examined through six core domains; timeliness, safety, effectiveness, equity, patient-centeredness and efficiency according to the WHO framework on quality of health services. ^10^ To ensure clarity and consistency, operational definitions of the terms used in this study are presented in Table 1.

**Table 1.** Operational definitions: The table below contains the operational definitions of key terms that would be used in this work.

Term	Definitions
Maternal Health services	Refer to the range of health services provided to women during pregnancy, childbirth, and the immediate postpartum period with the aim of promoting and maintaining the physical, mental, and social well-being of the mother. These services include antenatal care, intranatal care-skilled care during labour and delivery, emergency obstetric care, and immediate postpartum care delivered through the formal health system. ^10^
Quality of maternal health services	The extent to which health care services are provided to individuals and patient populations to improve desired health outcomes. To achieve this, health care must be safe, effective, timely, efficient, equitable, and people centered. • Safe – delivering health care that minimizes risks and harm to service users, including avoiding preventable injuries and reducing medical errors. • Effective – providing services based on scientific knowledge and evidence-based guidelines. • Timely – reducing delays in providing and receiving health care. • Efficient – delivering health care in a manner that maximizes resource use and avoids waste. • Equitable – delivering health care that does not differ in quality according to personal characteristics such as gender, race, ethnicity, geographical location or socioeconomic status. • People-centered – providing care that takes into account the preferences and aspirations of individual service users and the culture of their community. ^21^
Access to maternal health service	Access to maternal health services refers to a woman’s ability to reach and use appropriate maternal health services when needed, including physical accessibility, availability of services, and actual utilization of care. ^22^
Low- and middle-income countries	Countries classified by the World Bank as low-income, lower-middle-income, or upper-middle-income based on gross national income (GNI) per capita in the year the study was conducted. ^23^

Maternal mortality and morbidity remain important health outcomes, particularly in LMICs, where most global maternal deaths occur. ^12^ Studies conducted in LMICs have reported delayed care seeking, reduced antenatal attendance, lower rates of facility-based delivery, disrupted referral pathways, shortages of skilled health workers, and compromised facility readiness during flood events. ^13^ ^,^ ^14^ Given the diversity of study designs, settings, and methodological approaches in this field, a scoping review will be conducted to systematically map the available evidence on how heavy rainfall and flooding affect the accessibility and quality of maternal health services in LMICs. The findings of this scoping review will inform future primary research and support policy development and planning efforts, aimed at strengthening climate-resilient maternal health systems and improving maternal health outcomes in vulnerable settings.

A conceptual framework has been developed to illustrate the pathways through which heavy rainfall and flooding affect maternal health service delivery in LMIC ( Figure 1).

**Figure 1.**
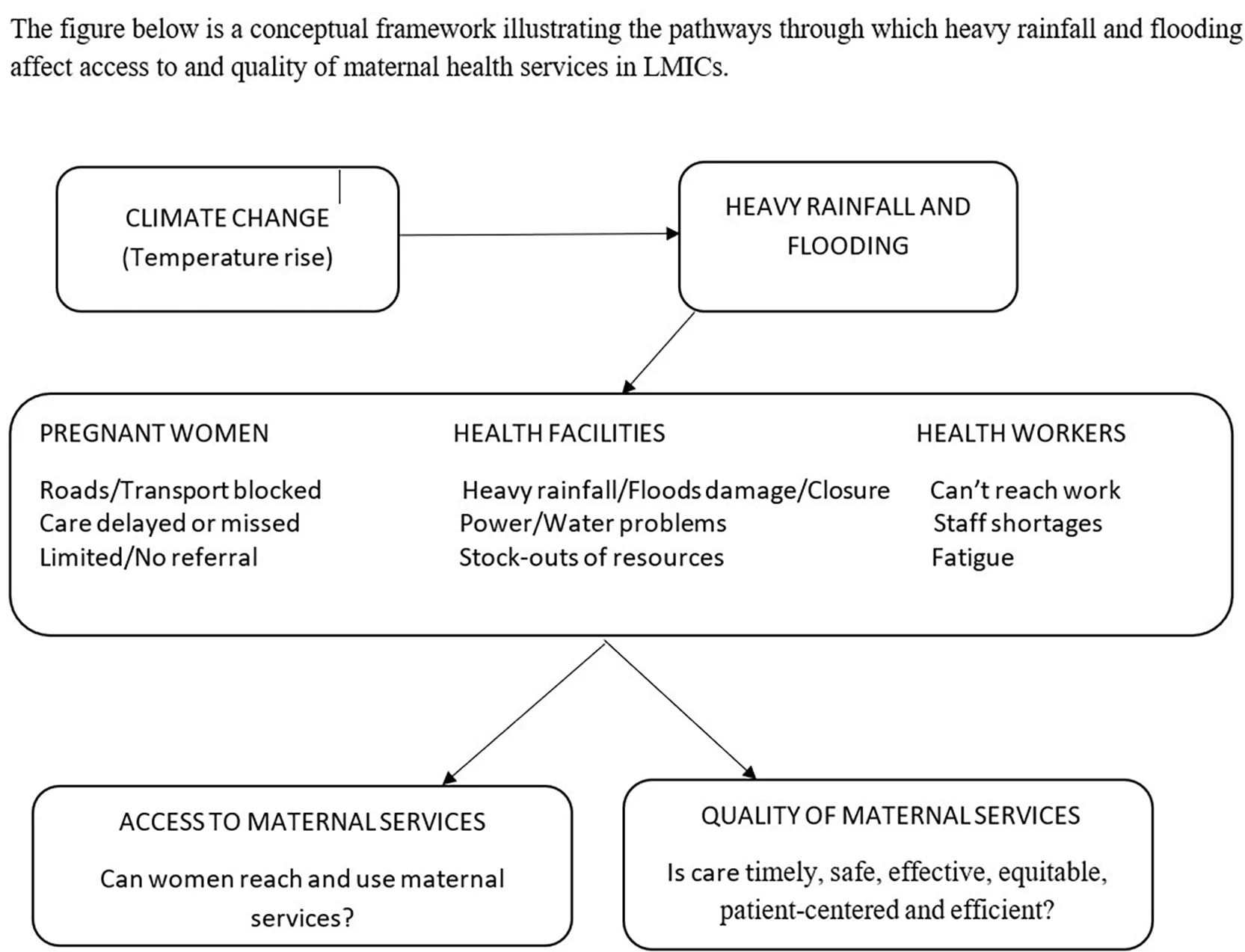
Conceptual framework illustrating the pathways through which heavy rainfall and flooding affect maternal health service delivery in LMICs. The framework shows how climate-related events can disrupt transportation systems, health facility functionality, and healthcare workforce availability, thereby affecting access to maternal health services and the quality of care provided during pregnancy and childbirth.

The framework highlights how climate-related events disrupt transportation systems, health facility functionality and healthcare workforce availability, ultimately influencing both access to maternal health services and the quality of care provided.

### Objective

**Broad Objective:** To map and synthesize existing evidence on how heavy rainfall and flooding affect accessibility and quality of maternal health service delivery during pregnancy and childbirth in LMICs.

**Research Question**: How does heavy rainfall and flooding as an extreme weather event affect accessibility and quality of maternal health services during pregnancy and childbirth in LMICs?

**Specific objectives:**

1. To assess how heavy rainfall and flooding affect access to key maternal health services during pregnancy and childbirth in LMICs.
2. To examine the effects of heavy rainfall and flooding on the quality of maternal health service delivery during pregnancy and childbirth in LMICs.

## Methods

This exploratory scoping review will follow the JBI Scoping Review methodology. ^15^ It will be an exploratory scoping review that will map and describe existing evidence on how heavy rainfall and flooding affect maternal health service delivery during pregnancy and childbirth in LMIC.

### Patient and Public Involvement

Patient and public were not involved in the development of this scoping review protocol. This study involves synthesis of evidence from previously published literature and does not involve primary data collection.

### Eligibility Criteria

The eligibility criteria for this scoping review will be guided by the Population–Concept–Context (PCC) framework. ^15^ The PCC framework is defined to align closely with the review objectives and research question by specifying the target population, the climate-related exposures of interest, and the contextual setting. We will therefore capture relevant articles on how heavy rainfall and flooding affect maternal health service delivery in LMICs, while excluding studies outside the defined scope in terms of population, exposure, outcomes, setting, study design, and publication characteristics. A full summary of the inclusion and exclusion criteria is presented in Table 2.

**Table 2.** Inclusion and exclusion criteria.

Inclusion criteria	Exclusion criteria
• Studies involving pregnant women, women in labour/childbirth, or early postpartum women in LMICs. • Studies involving health workers or health facilities providing maternal health services. • Studies that examine heavy rainfall and flooding events. • Studies reporting effects of rainfall and flooding on maternal health service delivery (access or quality). • Studies conducted in low- and middle-income countries. • Any empirical study design (quantitative, qualitative, mixed methods) and relevant reports. • Review studies • Published in English. • Published between 1990 and 2025.	• Studies focusing only on neonates, children, or general populations without maternal service delivery relevance. • Studies on climate hazards other than heavy rainfall or flooding (e.g., heatwaves, droughts) unless heavy rainfall and/or flooding is clearly included. • Studies reporting only clinical outcomes (e.g., mortality) without examining service delivery, access, or quality. • Studies conducted exclusively in high-income countries. • Editorials, commentaries, opinion pieces, protocols without results, and conference abstracts without full text. • Studies published before 1990 or not in English.

### Search Strategy

The scoping review search strategy will be designed in accordance with the PCC framework, in consultation with a research librarian, to ensure comprehensive identification of relevant literature search terms related to the population, concept, and context, and will be combined using Boolean operators “OR” and “AND”. Synonyms and related terms will be included to capture variations in terminology across disciplines and study designs. The key search terms and corresponding MeSH terms developed using the PCC framework are illustrated in ( Table 3). The full search strategy, including database-specific search strings and combinations of terms, is provided in ( Tables 4 and 5).

**Table 3.** Key search terms and corresponding MeSH terms developed using the PCC framework.

PCC Element	Core Concept	Keywords	MeSH terms
Population	Pregnant, Intranatal and early postpartum women	Pregnant, gravida, gestational, antenatal, prenatal, intrapartum, intranatal, labour, labor, childbirth, birth, delivery, postpartum, post-partum, maternal, postnatal, parturition	Pregnant Women; Pregnancy; Parturition; Delivery, Obstetric; Puerperium; Maternal Health Services
Population	Maternal health providers/facilities	Midwife, nurse, doctor, clinician, medical doctor, skilled birth attendant, health professional, obstetrician, health worker, health personnel, health facility, maternity ward, maternity unit, health facility	Health Personnel; Midwifery; Obstetrics, Hospitals, Maternity; Health Facilities
Concept	Heavy rainfall/flooding/climate related hazard	Floods, flash flood, river flood, coastal flood, heavy rain, intense rain, extreme rain, heavy precipitation, extreme precipitation, rainfall event, extreme weather	Floods; Rain; Precipitation; Natural Disasters; Climate Change; Extreme weather, extreme weather events, severe weather
Concept	Maternal health service delivery	maternal health service, maternity care, obstetric care, basic obstetric care, emergency obstetric care, comprehensive obstetric care, skilled birth attendance, facility-based delivery, facility birth, institutional delivery, ANC, antenatal care, prenatal care, postnatal care, post-partum care, continuity of care, health service, service disruption, health system	Maternal Health Services; Obstetric Surgical Procedures; Prenatal Care; Postnatal Care; Delivery of Health Care; Health Services Accessibility; Continuity of Patient Care; Health Services
Concept	Access	access, accessibility, health service access, access to care, care seeking, care-seeking, help seeking, delayed care, missed visit*, service utilization, service utilisation, healthcare utilization, health care utilization, geographic access, physical access, transport barrier, road access, referral delay, referral breakdown, barriers to care, service disruption, service interruption, facility closure, reaching care, delay in reaching care	Health Services Accessibility
Concept	Quality	quality of care, quality improvement, care quality, health care quality, maternal care quality, obstetric care quality, safe care, patient safety, service safety, safety of care, quality standards, quality indicators, quality measures, timeliness of care, timely care, delays in care, delay in receiving care, waiting time, efficiency, service efficiency, health system efficiency, continuity of care, continuity of services, disrupted continuity, referral quality, facility readiness, facility preparedness, service readiness, health facility readiness, emergency obstetric readiness, availability of skilled staff, staff shortages, equipment availability, supply availability, drug shortages, service capacity, quality challenges, compromised quality, poor quality care	Quality of Health Care; Patient Safety; Efficiency, Organizational; Continuity of Patient Care; Referral and Consultation
Context	Low- and middle-income countries	low-income country, middle-income country, low- and middle-income country, LMIC, developing country, Low- and middle-income populations, low- and middle-income economies, low- and middle-income nations, low-income economies, low-income populations, middle income economies, middle income populations, resource-limited setting, resource-constrained setting, and Specific **regional terms**	Developing Countries; region-specific headings such as Africa South of the Sahara; Asia, Southeastern; Latin America

**Table 4.** PubMed search strategy 1.

Name of the database: PubMed (1) - on 4 ^th^, December 2025		
	Search query	Number of articles hit
**Search term category 1- Population** Combined both population categories with OR.	(Pregnant OR gravida OR gestational OR antenatal OR prenatal OR intrapartum OR intranatal OR labour OR labor OR childbirth OR delivery OR postpartum OR post-partum OR maternal OR postnatal AND (1990:3000/12/12[pdat])) OR (Midwife OR nurse OR obstetrician OR “health worker” OR “health personnel” OR “health facility” OR “maternity ward” OR “maternity unit”	**4,720,253**
**Search term category 2** **Concept 1- exposure (heavy rainfall and flooding)** Combined all three with OR Maternal health services OR access OR quality	Flood* OR “flash flood” OR “river flood” OR “coastal flood” OR “heavy rain” OR “intense rain” OR “extreme rain” OR “heavy precipitation” OR “extreme precipitation” OR “rainfall event” OR “extreme weather”	**35822**
**Search term category 3** **Concept 2- (access and quality of maternal health services)**	(((“maternal health service” OR “maternity care” OR “obstetric care” OR “emergency obstetric care” OR “skilled birth attendance” OR “facility-based delivery” OR “facility birth” OR “institutional delivery” OR ANC OR “antenatal care” OR “prenatal care” OR “postnatal care” OR “post-partum care” OR “continuity of care” OR “health service” OR “service disruption” OR “health system” AND (1990:3000/12/12[pdat])) OR (access OR “health service access” OR “geographic access” OR “physical access” OR “care seeking” OR “delayed care” OR “three delays” OR “service utilization” OR “service use” OR “service disruption” AND (1990:3000/12/12[pdat]))) OR (“Timely care” OR timely OR “quality of care” OR “service quality” OR “patient safety” OR “facility readiness” OR efficient OR “continuity of care” AND (1990:3000/12/12[pdat])) AND (1990:3000/12/12[pdat]))	**3,016,326**
**Search term category 4 (1 AND 2 AND 3)**		**1622**
**Search term category 5** **Context- LMIC**	“low-income country” OR “middle-income country” OR “low- and middle-income country” OR LMIC OR “developing country” OR “low- and middle-income populations” OR “low- and middle-income economies” OR “low- and middle-income nations” OR “low-income economies” OR “low-income populations” OR “middle income economies” OR “middle-income populations” OR “resource-limited setting” OR “resource-constrained setting” OR **reginal terms according to the world bank classification** ^23^	**4,614,455**
**Search term category 6 (4 AND 5)**	Final string after combining it all.	**563**

**Table 5.** Search Strategy 2: After adding more terms on Access and Quality.

Name of the database: PubMed – on 8 ^th^, December 2025		
**Search term category 1- population** Combined both population categories with OR.	(Pregnant OR gravida OR gestational OR antenatal OR prenatal OR intrapartum OR intranatal OR labour OR labor OR childbirth OR delivery OR postpartum OR post-partum OR maternal OR postnatal OR parturition AND (1990:3000/12/12[pdat])) OR (Midwife OR nurse OR doctor OR “medical doctor” OR clinician OR “medical doctor” OR “skilled birth attendant” OR “health professionals” OR obstetrician OR “health worker” OR “health personnel” OR “health facility” OR “maternity ward” OR “maternity unit” OR “health facility” AND (1990:3000/12/12[pdat])) Filters: from 1990–3000/12/12	**5,539,849**
**Search term category 2** **Concept 1- exposure (heavy rainfall and flooding)**	Flood* OR “flash flood” OR “river flood” OR “coastal flood” OR “heavy rain” OR “intense rain” OR “extreme rain” OR “heavy precipitation” OR “extreme precipitation” OR “rainfall event” OR “extreme weather”	**35,848**
**Search term category 3** **Concept 2- (access and quality of maternal health services)** Combined all the three with OR Maternal health services OR access OR quality	((“maternal health service” OR “maternity care” OR “obstetric care” OR “emergency obstetric care” OR “comprehensive obstetric care” OR “skilled birth attendance” OR “facility-based delivery” OR “facility birth” OR “institutional delivery” OR ANC OR “antenatal care” OR “prenatal care” OR “postnatal care” OR “post-partum care” OR “continuity of care” OR “health service” OR “service disruption” OR “health system” AND (1990:3000/12/12[pdat])) OR (access OR accessibility OR “health service* access” OR “access to care” OR “care seeking” OR “care-seeking” OR “help seeking” OR “delayed care” OR “missed visit*” OR “service utilization” OR “service utilisation” OR “healthcare utilization” OR “health care utilization” OR “geographic access” OR “physical access” OR “transport barrier*” OR “road access*” OR “referral delay*” OR “referral breakdown*” OR “barriers to care” OR “service disruption*” OR “service interruption*” OR “facility closure*” OR “reaching care” OR “delay in reaching care” AND (1990:3000/12/12[pdat]))) OR (“quality of care” OR “quality improvement” OR “care quality” OR “health care quality” OR “maternal care quality” OR “obstetric care quality” OR “safe care” OR “patient safety” OR “service safety” OR “safety of care” OR “quality standards” OR “quality indicators” OR “quality measures” OR “timeliness of care” OR “timely care” OR “delays in care” OR “delay in receiving care” OR “waiting time” OR efficiency OR “service efficiency” OR “health system efficiency” OR “continuity of care” OR “continuity of services” OR “disrupted continuity” OR “referral quality” OR “facility readiness” OR “facility preparedness” OR “service readiness” OR “health facility readiness” OR “emergency obstetric readiness” OR “availability of skilled staff” OR “staff shortages” OR “equipment availability” OR “supply availability” OR “drug shortages” OR “service capacity” OR “quality challenges” OR “compromised quality” OR “poor quality care” AND (1990:3000/12/12[pdat]))	**2,904,374**
**Search term category 4 (1 AND 2 AND 3)**		**1,629**
**Search term category 5** **Context- LMIC**	“low-income country” OR “middle-income country” OR “low- and middle-income country” OR LMIC OR “developing country” OR “low- and middle-income populations” OR “low- and middle-income economies” OR “low- and middle-income nations” OR “low-income economies” OR “low-income populations” OR “middle income economies” OR “middle-income populations” OR “resource-limited setting” OR “resource-constrained setting” OR reginal terms according to the world bank classification. ^23^	**4,614,455**
**Search term category 6 (4 AND 5)**	Final string after combining it all.	**559**

### Information Sources

A comprehensive search will be conducted in MEDLINE, Global Health, and Web of Science, as well as relevant grey literature sources. The search will be limited to studies published in English between 1990 to 2025 as guided by the Kyoto Protocol establishing the base year for monitoring greenhouse gas reductions as 1990. ^16^ Reference list of included studies and relevant reviews will be screened to identify additional relevant publications. An initial search strategy, which will be refined during the review, has been developed ( Tables 4 & 5).

### Study Selection and Screening

Retrieved articles will be imported to Covidence software, ^17^ where de-duplication will be performed automatically. The title and abstract screening will be conducted by two independent reviewers using the predefined inclusion and exclusion criteria. Articles deemed potentially eligible will proceed to full text screening, which will be conducted by two reviewers independently (VB) ^1^ and (MN) ^1^. Any disagreements at either stage will be resolved through discussion and where consensus cannot be reached, a third reviewer will be consulted. The study selection process will be documented and summarized using the Preferred Reporting Items for Systematic Reviews and Meta-Analyses (PRISMA) flow diagram. ^18^

### Data Charting and Extraction

Relevant data will be extracted using a pre-developed data charting template in Covidence and then exported to Excel for analysis. Extracted data will include aspects such as study characteristics, type of heavy rainfall or flooding events, maternal health service components examined, and key findings related to access and quality of maternal health service delivery. Data extraction will be conducted by one reviewer (VB) ^1^, with a second reviewer independently (CW) ^1^ checking a 20% sample of extracted studies for consistency and accuracy [1]. Any discrepancies identified will be discussed and resolved by consensus. A sample data extraction table is included in Table 6.

**Table 6.** Data extraction table (A sample data extraction table detailing the variables to be collected).

**Bibliography**	Author(s) Year of Publication Study Title
**Country/setting**	Country where the study was conducted and classification as an LMIC
**Study design**	Study design (e.g., cross-sectional, cohort, case-control, qualitative, mixed methods)
**Study population**	Characteristics of participants (e.g., pregnant women, postpartum women, healthcare providers)
**Exposure (rainfall/flooding)**	Type of climate event examined (e.g., flooding, heavy rainfall, extreme precipitation)
**Maternal health service outcome**	Type of maternal health service examined (e.g., antenatal care, skilled birth attendance, facility delivery, immediate postnatal care)
**Key findings**	Main results related to the impact of flooding/heavy rainfall on maternal health services
**Study limitations**	Limitations reported by the authors

### Synthesis and Presentation of Results

Extracted data will be cleaned and organized using Microsoft Excel prior to synthesis. Findings will be synthesized descriptively and narratively. For Qualitative data, we will employ both deductive and inductive thematic analysis. Results will be grouped according to key dimensions of maternal health service delivery, including access and quality of care, with quality further organized by domains such as timeliness, safety, effectiveness, equitability, patient-centeredness and efficiency. The results will be presented in a combination of tables and narrative summaries to map the range and characteristics of the existing evidence. Reporting of the review will follow the Preferred Reporting Items for Systematic Reviews and Meta-Analyses extension for Scoping Reviews, PRISMA-ScR, ^18^ where appropriate, findings will be presented by geographic region, type of flooding or rainfall event, and maternal health service components to enhance clarity and interpretability.

#### Ethics and Dissemination

This scoping review is nested within an ongoing Novel Extreme Weather and Risk Insurance for Kenya, NEWRISK project. NEWRISK has received ethical approval from the Scientific and Ethics Review Unit, Kenya Medical Research Institute (KEMRI/SERU/CGMR-C/315/4971). The findings of this review will be disseminated through publication in a peer-reviewed journal and presentation at relevant academic and policy forums. The results will contribute to understanding how heavy rainfall and flooding affect maternal health service delivery in low- and middle-income countries and may inform future research, policy development, and health system planning aimed at strengthening climate-resilient maternal health services.

#### Protocol registration

This protocol is registered on the Open Science Framework (OSF) under the Creative Commons Attribution-ShareAlike 4.0 International (CCBY-SA 4.0) license. ^19^ All protocol metadata and methodological details are thus publicly available. DOI: https://doi.org/10.17605/OSF.IO/E9ZFC. ^19^

## Data Availability

No primary data is associated with this article. All data used in this study will be derived from previously published literature and will be cited within the review. Harvard Dataverse. PRISMA-ScR Checklist for Heavy Rainfall and Flooding Effects on Maternal Health Service Delivery During Pregnancy and Childbirth in LMIC. https://dataverse.harvard.edu/dataset.xhtml?persistentId=doi:10.7910/DVN/2HDGOU. ^20^ This project contains the following extended data:
• PRISMA-ScR_Checklist (Completed PRISMA Extension for Scoping Reviews checklist accompanying the protocol.) Data is available under the terms of the CC BY 4.0. This protocol has been developed in accordance with the Preferred Reporting Items for Systematic Review and Meta-Analysis Protocols extension for scoping reviews (PRISMA-ScR) guideline. A completed PRISMA-ScR checklist is available as extended data in the Harvard Dataverse repository. ^20^

## Footnotes

^1^
VB, Valentine Bahati, a Post-graduate diploma fellow sponsored by IDeAL and is the lead reviewer who will conduct all data extraction and full text screening, MN, Moriasi Nyanchoka, a Health Service Unit Researcher will conduct full text screening as a second reviewer, and CW, Conrad Wanyama, a DPhil fellow, will provide quality checks and supervision, while assessing the data extraction process for accuracy and reliability. All are affiliated with the KEMRI-Wellcome Trust Research Programme.
