# Supplementary material for: Heavy Rainfall and Flooding Effects on Maternal Health Service Delivery During Pregnancy and Childbirth in LMICs: A scoping review protocol
Source: NIHR Open Res. 2026 Aug 18;6:121. [Version 1] doi: 10.3310/nihropenres.14311.1 (PMC13627932; doi:10.3310/nihropenres.14311.1)
Supplement: Peer review history

# Reviewer report: Reviewer response for version 1

**DOI:** 10.3310/nihropenres.15594.r42129

**Published:** 2026-09-30

**Authors:** Dell Saulnier

**Affiliations:** 1 Karolinska Institutet, Stockholm, Sweden

The protocol is for a scoping review to map the effects of heavy rainfall and flooding on the accessibility and quality of maternal health services in LMICs. Using the JBI methodology, the review will include qualitative, quantitative and mixed-methods scientific studies, reports, and evaluations between 1990 and 2025. The data will be analyzed for patterns and mechanisms of service disruption.

 In addition to the comments from first reviewer, please find some additional suggestions for the review:

 1. How will you integrate the quantitative and qualitative data in the synthesis and results? Please clarify how the data will be analyzed so that the results complement each other.

 2. There is a strong assumption that floods and heavy rainfall are always disruptive or destabilizing to health systems. It is worthwhile to consider how any potentially positive effects will be treated in the analysis and data extraction.

 3. Related to above, the authors should consider what definitions they are using for floods and rainfall and how they will conceptualize the effects in their analysis. For instance, it is likely there will be a difference in settings that do not regularly experience floods or between flood disasters and smaller-scale events. Please explain how the exposures will be grouped and approached for the data analysis.

 4. Access is defined here as a woman’s ability to reach and use appropriate services when needed and the emphasis in the protocol and conceptual framework is on physical access (e.g. blocked roads). It would be worthwhile to expand the framework to make the potential pathways to impact clearer before data analysis, especially if the analysis will focus on the mechanisms that affect access and quality. For instance, studies may surface other reasons for changes in access, such as other priorities during a flood, knowledge of whether services have changed, lack of time, etc., that are either exacerbated or caused by the flood.

 5. Please clarify the time period for effects following the event. How will the authors address potential delays in access, for example, women who delay ANC but still access it after the flood ends or adequate versus complete ANC following a flood?

 6. In the eligibility criteria, studies will be included if they “involve pregnant women, women in labour/childbirth or early postpartum women”. Does this mean that studies will only be included if women participated who were pregnant, delivering, or early postpartum, or does it mean any studies that looked at women at these times?

 7. Please clarify how effects on access and quality, by the dimensions proposed in the tables, and their relationship to services will be captured in the data sheet. For instance, how will mechanisms be captured in the extracted data? The data sheet could be more fully explained.

Is the study design appropriate for the research question?

Yes

Is the rationale for, and objectives of, the study clearly described?

Yes

Are sufficient details of the methods provided to allow replication by others?

Partly

Are the datasets clearly presented in a useable and accessible format?

Not applicable

Reviewer Expertise:

Floods, health systems

I confirm that I have read this submission and believe that I have an appropriate level of expertise to confirm that it is of an acceptable scientific standard.

# Reviewer report: Reviewer response for version 1

**DOI:** 10.3310/nihropenres.15594.r42130

**Published:** 2026-09-16

**Authors:** Cara Schulte

**Affiliations:** 1 Climate Rights International, Berkeley, CA, USA

The protocol proposes a scoping review on the impacts of heavy rainfall and flooding on the accessibility and quality of maternal health service delivery - with a focus on the pregnancy and birth period - in LMICs. The paper will use the JBI methodology for scoping reviews and will report findings in alignment with the PRISMA-ScR. The researchers will use pre-defined terms to search MEDLINE, Global Health, Web of Science, and relevant gray literature for English-language sources published between 1990 and 2025.  

 Please find my questions and suggestions below:

- Please explain the decision to exclude postpartum and neonatal care from this review. How might the pathways be different in these contexts? What is the value of excluding this critical period? I might suggest extending the search to one year following delivery. 
- Consider adding “perinatal” to the population search terms. 
- Consider including “doula” or “community health worker” in the population search terms. 
- Might consider adding “childbirth” to the maternal health service delivery search terms. Was the decision to exclude this because it brought back irrelevant studies? 
- For search term five, I would consider explicitly including the country names of all LMICs, as not all local studies will be tagged with terms related to economic classification, but all studies will list the country name. 
- Please clarify how relevant gray literature will be identified. 
- Please clarify how heavy rainfall and/or flooding will be defined in the context of this scoping review - assume you will capture all definitions as referenced in each study captured? 
- Please clarify whether you will include financial barriers to healthcare access during periods of heavy rainfall or flooding? For example, if transportation becomes less available and therefore more expensive, will these types of barriers be captured? 
- Please clarify whether you will include rain and/or flood-related disruptions to medication and healthcare material supply chains? If so, please include relevant search terms.  
- Please clarify whether there is a specific period following the extreme weather event through which you will consider healthcare service disruptions relevant to the study? For example, will you limit findings to disruptions immediately following the rain or flood or for up to one year after the event?

Is the study design appropriate for the research question?

Yes

Is the rationale for, and objectives of, the study clearly described?

Yes

Are sufficient details of the methods provided to allow replication by others?

Yes

Are the datasets clearly presented in a useable and accessible format?

Not applicable

Reviewer Expertise:

Climate change, perinatal health, extreme heat, public health, human rights

I confirm that I have read this submission and believe that I have an appropriate level of expertise to confirm that it is of an acceptable scientific standard.
